# Implementation of continuous renal replacement therapy with regional citrate anticoagulation on a surgical and trauma intensive care unit: impact on clinical and economic aspects—an observational study

**DOI:** 10.1186/s40560-015-0102-7

**Published:** 2015-07-30

**Authors:** Sebastian Hafner, Wolfgang Stahl, Theresa Fels, Karl Träger, Michael Georgieff, Martin Wepler

**Affiliations:** Department of Anaesthesiology, Ulm University, Albert-Einstein-Allee 23, 89081 Ulm, Germany; Department of Cardioanaesthesiology, Ulm University, Albert-Einstein-Allee 23, 89081 Ulm, Germany

**Keywords:** Citric acid, Heparin, Renal replacement therapy, Acute kidney injury, Cost-benefit analysis

## Abstract

**Background:**

Regional citrate anticoagulation (RCA) is being increasingly used during continuous renal replacement therapy (CRRT) in intensive care units as an alternative to systemic heparin anticoagulation. However, due to its availability in a variety of solutions and dialysis systems, RCA is still considered a complex intervention, possibly leading to confusion and pitfalls in everyday practice. We therefore tested retrospectively if the introduction of RCA as a new anticoagulation strategy for CRRT was feasible and had not negatively impacted efficacy, safety, metabolic stability, filter lifetime, and cost-effectiveness compared to well-established systemic heparin.

**Methods:**

This observational, retrospective study was performed on a non-cardiac surgical and trauma intensive care unit (ICU) in a university hospital. All charts of patients receiving one of the CRRT techniques from May 2006 to April 2010 were reviewed. The first 60 consecutive patients receiving CRRT with regional citrate anticoagulation after its implementation in February 2008 (continuous veno-venous haemodialysis, Multifiltrate® with integrated CiCa® system, AV 1000 S® filter, *n* = 60) were included in the study. The last 50 consecutive patients with systemic heparin anticoagulation therapy (continuous veno-venous haemodiafiltration, PRISMAFLEX®, AN69® filter, *n* = 50), treated immediately before the introduction of RCA, were used as a historic control group.

**Results:**

Both treatment modalities were effective in terms of uraemia control. Patients in the citrate group presented with significantly higher pH levels, lower ionized calcium levels, and higher sodium levels compared with the heparin treated group, however, without notable adverse clinical events. Interestingly, mean circuit lifetime was significantly longer in the citrate group (48.6 ± 24.2 h vs. 18.8 ± 13.5 h; *p* < 0.0001). Both treatment modalities were cost-effective.

**Conclusions:**

Our results suggest that the implementation of regional citrate anticoagulation was safe and effective. Due to the retrospective design of the study and inherent limitations therein concerning several baseline parameters, i.e. different filters, modes of dialysis, and flow parameters not having been standardized, we were unable to draw a causative effect relationship. Nonetheless, our results warrant further study.

## Background

Acute kidney injury (AKI) and renal failure (ARF) are the major challenges during critical illness and represent a strong and independent risk factor for mortality [1]. On intensive care units, the incidence of AKI reaches about 30 % [2]. Continuous renal replacement therapy (CRRT) is a common treatment modality of ARF in critically ill patients [3]. To prevent clotting of the extracorporeal circuits, anticoagulation is required in most cases. Regional citrate anticoagulation (RCA) is increasingly used during CRRT on intensive care units as an alternative to systemic heparin due to advantages concerning the risk of bleeding and heparin-induced thrombocytopenia [4–7].

Citrate acts as an anticoagulant in the extracorporeal system through chelation of ionized calcium. Before the blood re-enters systemic circulation, calcium is replaced and the systemic coagulation cascade is maintained. Citrate is partially removed by filtration or dialysis and rapidly metabolized in the liver or other tissues. Since citrate is metabolized to bicarbonate, effects on the acid-base status and a trend towards metabolic alkalosis are common. Favourable effects on the inflammatory response in septic patients are discussed, as citrate may act as an anti-inflammatory agent as well [8, 9].

CRRT with regional citrate anticoagulation has to compete with well-established systemic heparin anticoagulation. However, due to its availability in a variety of solutions and dialysis systems, RCA is still considered a complex intervention, possibly leading to confusion and pitfalls in everyday practice [10]. We therefore tested retrospectively if the introduction of RCA as a new anticoagulation strategy for CRRT was feasible and had not negatively impacted efficacy, safety, metabolic stability, filter lifetime, and cost-effectiveness compared to well-established systemic heparin.

## Methods

### Subjects

This observational, retrospective study was performed on a 16 bed mixed non-cardiac surgical and trauma intensive care unit (ICU) in a university hospital after permission of the research ethics committee of Ulm University (“Ethikkommission der Universität Ulm”, reference number 54–14) according to the Helsinki Declaration. Due to the retrospective study design, no informed consent of an individual patient was needed. Patients with acute kidney injury according to the RIFLE criteria [11] and an indication for CRRT were included, independent of the reason for renal failure. Inclusion criteria were oligoanuria (<100 ml/24 h), excessive increase of serum creatinine and urea, hyperhydration with pulmonary edema not responsive to diuretics despite adequate blood pressure, or increase of serum potassium > 5.5 mmol/l due to oligoanuria. A period of CRRT of at least 3 days as well as the availability of all data sets concerning therapy on ICU was mandatory for inclusion in the study.

In 2008, citrate anticoagulation was established as a new anticoagulation strategy for continuous renal replacement therapy on a non-cardiac surgical and trauma ICU in a university hospital. Prior to this, heparin was used for systemic anticoagulation during CRRT. From May 2006 to December 2007, 50 consecutive patients were retrospectively analysed for the systemic heparin group. After a training and introduction period of 4 weeks, regional citrate anticoagulation was established as the sole mode of anticoagulation. Sixty consecutive patients receiving citrate anticoagulation from February 2008 through April 2010 were included in the study. Patient charts of every patient receiving one of the CRRT techniques from May 2006 to April 2010 were reviewed. An Access database (Microsoft Corporation, Redmond, USA) was created to collect anonymously demographic data such as age, weight, reasons for admission, and duration of stay on the ICU, among others. Comorbidities, pre-existing medication, the renal status before initiation of CRRT, as well as the Sequential Organ Failure Assessment (SOFA) score were also evaluated. Flow rates and period of CRRT, filter lifetime of the extracorporeal systems, and metabolic parameters were collected to analyse both treatment groups.

### Methods

**Fig. 1:**
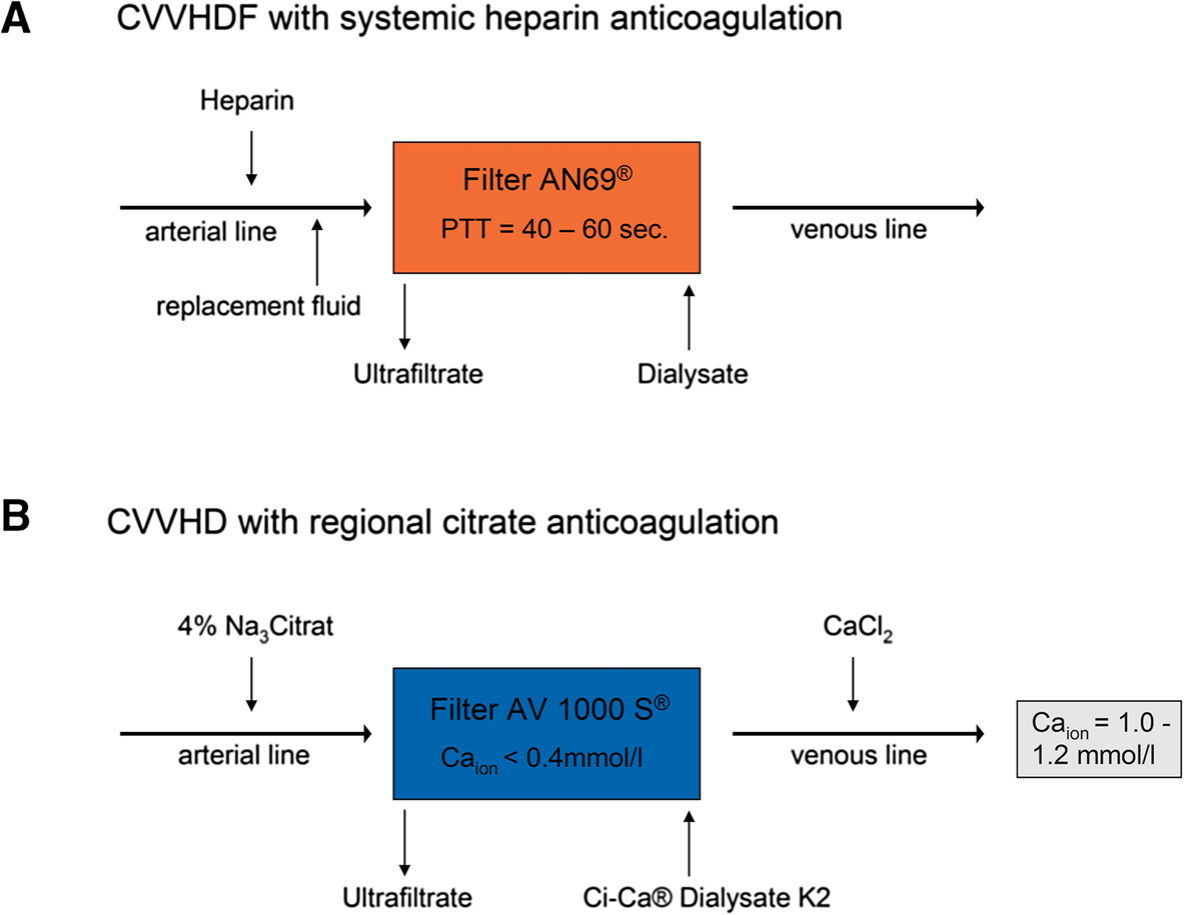
Treatment modalities used in the study. *CVVHDF* continuous veno-venous haemodiafiltration, *CVVHD* continuous veno-venous haemodialysis, *pTT* partial thromboplastin time, *sec* seconds, *Ca*_*ion*_ ionized calcium

Citrate CRRT was performed using commercially available equipment and solutions (Multifiltrate® with integrated CiCa® system, AV 1000 S® filter kit, CiCa® Dialysate K2, sodium citrate 4 %, and 0.5 M CaCl_2_ solution; Fresenius Medical Care, Bad Homburg, Germany; Table 1). As a mode of CRRT, continuous veno-venous haemodialysis (CVVHD) with regional citrate anticoagulation was chosen (Fig. 1) due to the fact that continuous veno-venous haemodiafiltration (CVVHDF) was not available for the Multifiltrate® with regional citrate anticoagulation at the time of introduction in our ICU. To maintain stable metabolic and haemodynamic conditions, a standard protocol for adjustments of blood, dialysate, citrate, and calcium flow was used as described previously [6].

**Table 1:** Composition of all dialysate solutions used in the study

	CiCa® Dialysate K2	Lactasol®	Hemosol BO®
Sodium (mmol/l)	133	140	140
Potassium (mmol/l)	2	0	0
Calcium (mmol/l)	0	1.75	1.75
Magnesium (mmol/l)	0.75	0.75	0.5
Chloride (mmol/l)	116.5	105	109.5
Bicarbonate (mmol/l)	20	0	32
Lactate (mmol/l)	0	40	3
Glucose (g/l)	1	0	0

Heparin CRRT was performed with PRISMAFLEX® and the corresponding filter kits and fluid solutions (AN69® filter kit, Lactasol®, or Hemosol BO®; Gambro, Hechingen, Germany; Table 1) as continuous veno-venous haemodiafiltration (Fig. 1). The dosage of heparin was 1000 IU per hour, with a target of partial thromboplastin time (pTT) between 40 and 60 s.

To compare the metabolic effects of both techniques, laboratory findings were analysed in all patients. Metabolic acidosis was defined by low pH (<7.35), reduced base excess (<−3 mmol/l), and a normal or reduced p_a_CO_2_ (≤44 mmHg). Metabolic alkalosis was defined by increased pH (>7.45), increased base excess (>3 mmol/l), and a normal or increased p_a_CO_2_ (≥36 mmHg). Hypernatremia was defined as a rise in serum sodium to a value exceeding 145 mmol/l. Reimbursement for both techniques was calculated on procedure-related rates according to the German Diagnosis-Related Groups (G-DRG).

### Objectives

The objectives were to evaluate exploratively the efficacy, safety, metabolic stability, filter lifetime, and cost-effectiveness of regional citrate anticoagulation during CRRT in comparison with systemic heparin anticoagulation. The chosen parameter of efficacy was the reduction of urea during CRRT course, measured on each consecutive day. Safety and metabolic stability were determined by electrolyte concentrations and the acid-base status on each consecutive day during the CRRT course. Filter lifetime was documented for every single filter and collected in a database. Cost-effectiveness was calculated on reimbursement (according to G-DRG) and disbursement (materials only).

### Statistical analysis

Statistical analysis was performed using descriptive methods; moreover, the Mann-Whitney test, the chi-square test, the Spearman correlation, and the log-rank test were applied where appropriate. *p* < 0.05 was exploratively regarded as statistically significant. For statistical calculations, GraphPad Prism 5, version 5.04, was used (GraphPad Software Inc., La Jolla, USA).

## Results

### Study population

In total, we analysed 110 patients receiving CRRT with regional citrate or systemic heparin anticoagulation from May 2006 through April 2010. In the systemic heparin group, 50 consecutive patients receiving CRRT from May 2006 through December 2007 were analysed, compared to 60 consecutive patients in the regional citrate group from February 2008 to April 2010.

### Baseline and demographic data

Table 2 shows the baseline and demographic parameters of the study population. Baseline characteristics as well as the mean SOFA scores at beginning of CRRT were similar in both groups. Mean dose of renal replacement therapy, mean dialysate flow, and mean ultrafiltration flow significantly differed between groups. Hepatobiliary disorders were not an exclusion criterion for anticoagulation with citrate during CRRT; however, no cases with severe liver failure were treated with citrate during the study period (Table 2).

**Table 2:** Demographic and baseline parameters of the study population

Parameter	CVVHD—citrate	CVVHDF—heparin	*p* value
Number of patients (*n*)	60	50	
Gender (*n*, male)	44 (73 %)	36 (72 %)	n.s.
Age (years)	68 ± 12	69 ± 11	n.s.
Bodyweight (kg)	87 ± 21	86 ± 23	n.s.
Sepsis (*n*)	16 (27 %)	16 (32 %)	n.s.
Postoperative (*n*)	40 (66 %)	25 (50 %)	n.s.
Other reason for ICU admission (*n*)	4 (7 %)	9 (18 %)	n.s.
SOFA score at initiation of CRRT	10 ± 3	9 ± 3	n.s.
Creatinine before present illness (μmol/l)	195 ± 139	226 ± 252	n.s.
Creatinine at initiation of CRRT (μmol/l)	337 ± 133	343 ± 179	n.s.
Urea before present illness (mmol/l)	11 ± 9	10 ± 9	n.s.
Urea at initiation of CRRT (mmol/l)	23 ± 9	22 ± 9	n.s.
Mean platelet count during CRRT (G/l)	168 ± 108	175 ± 115	n.s.
Mean dose of CRRT (ml∙h^−1^∙kg body weight^−1^)	24.0 ± 6.2	20.7 ± 5.6	0.0001
Mean blood flow (ml/min)	102 ± 12.5	100 ± 2.8	n.s.
Mean dialysate flow (ml/h)	2035 ± 307	1133 ± 283	0.0001
Mean flow replacement fluid (ml/h)	–	563 ± 203	n.a.
Mean ultrafiltration flow (ml/h)	159 ± 55	170 ± 59	0.0001
Mechanical ventilation (*n*)	43 (72 %)	40 (80 %)	n.s.
Cirrhosis of the liver (*n*)	6 (10 %)	4 (8 %)	n.s.
Patients weaned from CRRT (*n*)	22 (37 %)	19 (38 %)	n.s.
Patients receiving intermittent dialysis at ICU discharge (*n*)	8 (13 %)	4 (8 %)	n.s.
Fatal outcome on ICU (*n*)	30 (50 %)	27 (54 %)	n.s.

*CRRT* continuous renal replacement therapy, *CVVHDF* continuous veno-venous haemodiafiltration, *CVVHD* continuous veno-venous haemodialysis, *SOFA* Sequential Organ Failure Assessment, *ICU* intensive care unit, *n.s.* not significant, *n.a*. not applicable

### Efficacy

Control of uraemia was evaluated on each consecutive day during CRRT course. The mean plasma urea baseline levels at the initiation of CRRT were 22.98 ± 9.26 mmol/l in the citrate group and 21.48 ± 9.26 mmol/l in the systemic heparin group. Reduction of urea levels on day 2 was equally effective in both groups (16.43 ± 5.71 mmol/l in the citrate group and 17.54 ± 7.03 mmol/l in the systemic heparin group, *p* = 0.43). On day 3 and day 4, reduction of urea levels was more pronounced in the citrate group with a statistically significant difference (12.70 ± 3.94 mmol/l vs. 16.67 ± 6.38 mmol/l on day 3 and 12.40 ± 4.39 mmol/l vs. 17.94 ± 7.30 mmol/l on day 4; *p* = 0.003 and *p* < 0.0001, respectively).

### Safety and metabolic stability

**Fig. 2:**
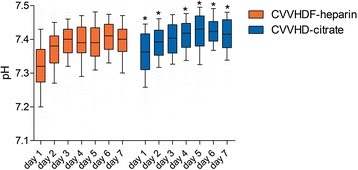
Course of pH during CRRT from day 1 to day 7 for all patients. Data are given as *box plots*, and *whiskers* represent the 10th/90th percentile. *p < 0.05 vs. heparin. *CRRT* continuous renal replacement therapy, *CVVHDF* continuous veno-venous haemodiafiltration, *CVVHD* continuous veno-venous haemodialysis

Both CRRT with regional citrate anticoagulation and with systemic heparin anticoagulation showed stable metabolic parameters with a trend towards a metabolic alkalosis in the citrate group, as pH was significantly higher in the citrate group during the complete CRRT course (*p* < 0.0001). In total, 14 of 60 patients (23 %) in the citrate group developed metabolic alkalosis compared to 2 out of 50 patients (4 %) in the heparin group (*p* = 0.0054). Metabolic acidosis occurred in 9 of 60 patients (15 %) in the citrate group, whereas 12 of 50 patients (24 %) developed metabolic acidosis in the heparin group (*p* = 0.33). In addition, base excess and bicarbonate were significantly higher in the citrate group on each consecutive day during CRRT course (*p* < 0.0006 and *p* < 0.00001, respectively). The course of pH during CRRT from day 1 to day 7 for all patients is shown in Fig. 2.

**Fig. 3:**
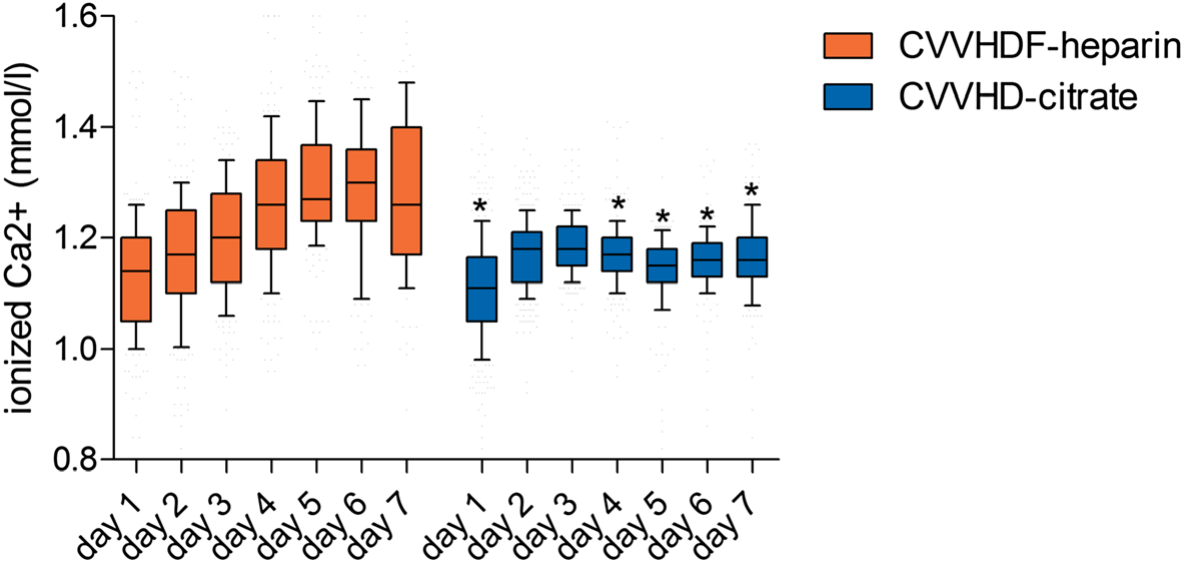
Course of ionized calcium during CRRT from day 1 to day 7 for all patients. Data are given as *box plots*, and *whiskers* represent the 10th/90th percentile. *p < 0.05 vs. heparin. *CRRT* continuous renal replacement therapy, *CVVHDF* continuous veno-venous haemodiafiltration, *CVVHD* continuous veno-venous haemodialysis

Values of ionized calcium during CRRT course were significantly lower in the citrate group (*p* < 0.0001). Nevertheless, hypocalcemia only occurred in 8 patients out of 60 patients (13 %) in the citrate group compared to 7 patients out of 50 patients (14 %) in the heparin group. No serious complications of hypocalcemia such as tetany or arrhythmia were observed. During the whole CRRT course, serum sodium was significantly higher in the citrate group (*p* < 0.0001). Hypernatremia only occurred in 8 patients out of 60 patients (13 %) in the citrate group, whereas 7 patients out of 50 (14 %) showed hypernatremia in the heparin group. Serum potassium was significantly higher in the heparin group during the whole CRRT course in all patients (*p* = 0.007). The course of ionized calcium during CRRT from day 1 to day 7 for both groups is shown in Fig. 3.

Two out of sixty patients in the citrate group showed signs of citrate accumulation, defined by a ratio of total serum calcium to ionized calcium >2.5. These derangements could be managed by a reduction of the blood flow and the citrate concentration according to a preset protocol [6]. Citrate CRRT could be continued in all patients.

### Filter lifetime

**Fig. 4:**
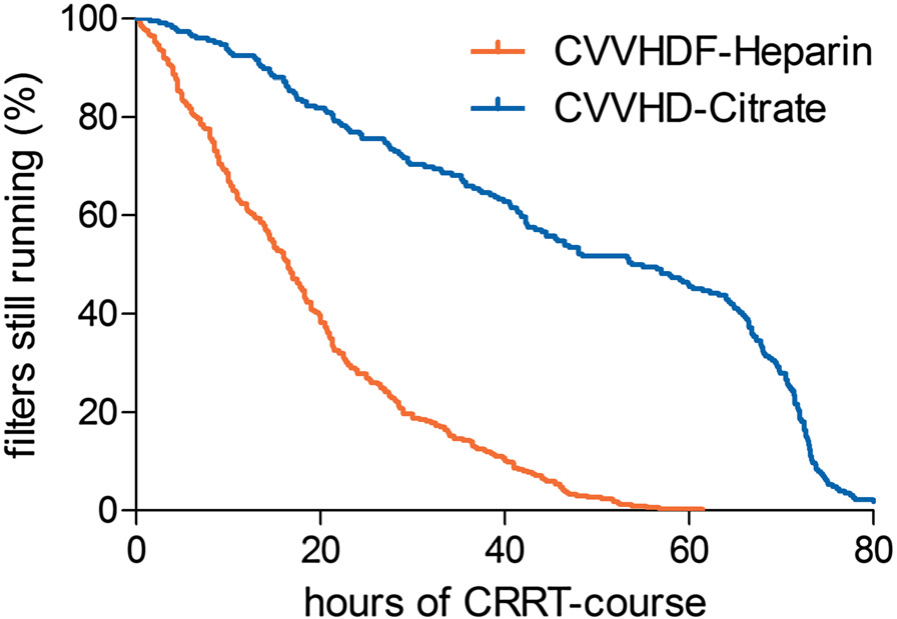
Filter lifetime of all filters used in the study. Log-rank test: *p* < 0.0001 citrate vs. heparin. *CRRT* continuous renal replacement therapy, *CVVHDF*, continuous veno-venous haemodiafiltration, *CVVHD* continuous veno-venous haemodialysis

The mean filter lifetime was 48.6 ± 24.2 h in the citrate group, compared to 18.8 ± 13.5 h in the heparin group (*p* < 0.0001). Because of a possible bias, data were also analysed after exclusion of the last filter running before cessation of CRRT. These data neither showed a significant difference between the citrate (50.1 ± 24.2 h) and the heparin groups (18.2 ± 13.0 h). In total, 226 filters were used in the citrate group, compared to 335 filters in the heparin group. This resulted in a median treatment time of 140.2 (68.8, 281.3) hours in the citrate group and a median treatment time of 88.5 (38.7, 176.8) hours in the heparin group (*p* = 0.0082). The median down times were 10 (0, 21) % of total dialysis time in the citrate group and 13 (5, 24) % of total dialysis time in the heparin group (*p* = 0.1286). In addition, we assessed a possible correlation between filter lifetime and the dose of dialysis: We could only find a weak inverse correlation between filter lifetime and mean dose of CRRT (Spearman *r* = −0.15; *p* = 0.008 in the citrate group and Spearman *r* = −0.26; *p* < 0.0001 in the heparin group, respectively). Filter patency of all filters is shown in Fig. 4.

### Cost-effectiveness

Reimbursement for both techniques was calculated on procedure-related rates according to G-DRG. While filter kits were more expensive in the citrate group, reimbursement increased due to a longer period of CRRT: The median costs per patient were 1440 (738, 3113) € in the citrate group and 863 (434, 1435) € in the heparin group for the complete CRRT course (*p* = 0.0017). The median reimbursement per patient was 2143 (770, 4679) € in the citrate group and 1553 (770, 2732) € in the heparin group (*p* = 0.42).

## Discussion

This study addresses the implications of RCA on efficacy, safety, metabolic stability, filter lifetime, and cost-effectiveness immediately after its implementation on a non-cardiac surgical and trauma ICU compared to systemic heparin anticoagulation. Our main findings were that (1) both treatment modalities were effective in terms of uraemia control, (2) patients in the citrate group revealed significantly higher pH levels, lower ionized calcium levels, and higher sodium levels without any adverse clinical events compared to the heparin-treated group, and (3) the mean circuit lifetime was significantly longer in the citrate group.

### Efficacy

Concerning the treatment efficacy of both methods, our study revealed—concordant to previously published data [12]—that both methods were able to clear urea from the systemic circulation in a similar manner. In addition, we showed a significant reduction of urea in the citrate group on day 3 and day 4 compared to the heparin group. These results may be associated to the difference in filter lifetime of both groups, as filters in the citrate group showed a statistically longer circuit lifetime. Furthermore, longer down times of the dialysis filters on day 3 and day 4 and a significantly lower dose of dialysis in the heparin group may have contributed to the significant urea reduction in the citrate group as well. Another aspect in this context is the influence of nutrition on blood urea levels: As indirect calorimetry was not routinely used and may not be validated during CRRT, an effect of a potential catabolic state during critical illness cannot be excluded.

### Safety and metabolic stability

The patients in both groups showed stable metabolic parameters. As already published by others [13, 14], analysis of acid-base status revealed a significantly higher incidence of metabolic alkalosis in the citrate group. Furthermore, analysis of calcium homeostasis showed a significant lower level of ionized calcium in the citrate group, with no significant difference in the incidence of hypocalcemia in both groups. Serum sodium was significantly higher in the citrate group with no significant difference in the incidence of hypernatremia. All metabolic derangements in the citrate group could be managed by adjustments of blood flow, citrate flow, calcium flow, and dialysate flow according to a preset protocol [6]. If this protocol was strictly followed, metabolic derangements could be monitored in time and controlled without any clinical consequences, which is in line with previously published data [6].

### Filter lifetime

The results concerning filter lifetime have to be interpreted with care due to the retrospective study design and limitations therein, i.e. different filters, mode and dose of dialysis, and flow parameters not having been standardized. In agreement with other trials [12–15], filter lifetime in the citrate group was significantly higher than in the heparin group. As the coagulation process is calcium dependent [16], longer filter patency in the citrate group could be explained by adequate inhibition of coagulation in the extracorporeal circuit. However, in some patients, we observed early filter or circuit clotting in the citrate group despite a postfilter calcium level <0.30 mmol/l. These findings may be due to non-coagulation-related factors, such as vascular access, design of the dialysis catheter, training of nurses, mode of CRRT, clogging, biocompatibility of the membranes, and filter size or filter type [17]. Moreover, undetected heparin-induced thrombocytopenia may contribute to this phenomenon [18]. In contrast to our study, two controlled trials could not confirm a significantly longer filter patency in the citrate group [9, 19]. Different modes and protocols of CRRT, as well as the use of low-molecular-weight heparin (LMWH) may be responsible for these findings [9].

### Cost-effectiveness

The analysis of cost-effectiveness showed that, despite more expensive filter kits, citrate CRRT was a cost-effective therapy. Given that longer filter lifetimes could have had a time-saving effect for ICU staff, regional citrate anticoagulation may have helped to decrease the workload on our intensive care unit. Unfortunately, these time-saving effects could not be analysed in this study due to the retrospective study design. A recently published multicenter study even described significantly lower costs for citrate anticoagulation and nursing staff for filter change [15]. However, this study was performed in the Netherlands with a different reimbursement situation compared to Germany [15]. Therefore, these results cannot be easily extrapolated to different countries.

### Bleeding risk

Recently, several meta-analyses [20, 21] summarized a significant reduction of the bleeding risk during CRRT using regional citrate anticoagulation in critically ill patients. Our study, in contrast to most studies included in these meta-analyses [9, 12–14, 19, 22], was performed on a mixed surgical and trauma ICU with a high percentage of postoperative patients at high bleeding risk. Therefore, it would have been of particular interest to analyse if there was a difference in terms of bleeding complications in the citrate group compared to systemic heparin. In addition to several methodological problems (change of the transfusion threshold during the study period, indication for transfusion of blood products up to the physician in charge), our study is clearly underpowered to answer this question.

### Limitations

Our study has several limitations: The retrospective, monocentric, observational “before and after” study design precludes the possibility of drawing causative conclusions. Furthermore, different filters, modes of dialysis, and flow parameters not having been standardized are additional confounding factors. Finally, heparin was not specifically excluded in the care of those patients undergoing citrate-based anticoagulation, e.g. for prophylaxis of deep vein thrombosis.

## Conclusions

Our results suggest that the implementation of regional citrate anticoagulation was safe and effective. Due to the retrospective design of the study and inherent limitations therein concerning several baseline parameters, i.e. different filters, modes of dialysis, and flow parameters not having been standardized, we were unable to draw a causative effect relationship. Nonetheless, our results warrant further study.

## Abbreviations

AKI: acute kidney injury
ARF: acute renal failure
CRRT: continuous renal replacement therapy
CVVHD: continuous veno-venous haemodialysis
CVVHDF: continuous veno-venous haemodiafiltration
G-DRG: German Diagnosis-Related Groups
ICU: intensive care unit
LMWH: low-molecular-weight heparin
pTT: partial thromboplastin time
RCA: regional citrate anticoagulation
RIFLE: risk of renal dysfunction, injury to the kidney, failure of kidney function, loss of kidney function, and end-stage kidney disease
SOFA: Sequential Organ Failure Assessment
